# Obesity, diet quality, physical activity, and the built environment: the need for behavioral pathways

**DOI:** 10.1186/s12889-016-3798-y

**Published:** 2016-11-10

**Authors:** Adam Drewnowski, Anju Aggarwal, Wesley Tang, Philip M. Hurvitz, Jason Scully, Orion Stewart, Anne Vernez Moudon

**Affiliations:** 1 Center for Public Health Nutrition, 1107 NE 45th St, University of Washington, Seattle, WA 98105 USA; 2 Urban Form Lab, 1107 NE 45th St, University of Washington, Seattle, WA 98105 USA; 3 University of Washington, Box 353410, Seattle, WA 98195 USA

**Keywords:** Built environment, Physical activity, Obesity, Diet quality

## Abstract

**Background:**

The built environment (BE) is said to influence local obesity rates. Few studies have explored causal pathways between home-neighborhood BE variables and health outcomes such as obesity. Such pathways are likely to involve both physical activity and diet.

**Methods:**

The Seattle Obesity Study (SOS II) was a longitudinal cohort of 440 adult residents of King Co, WA. Home addresses were geocoded. Home-neighborhood BE measures were framed as counts and densities of food sources and physical activity locations. Tax parcel property values were obtained from County tax assessor. Healthy Eating Index (HEI 2010) scores were constructed using data from food frequency questionnaires. Physical activity (PA) was obtained by self-report. Weights and heights were measured at baseline and following 12 months’ exposure. Multivariable regressions examined the associations among BE measures at baseline, health behaviors (HEI-2010 and physical activity) at baseline, and health outcome both cross-sectionally and longitudinally.

**Results:**

None of the conventional neighborhood BE metrics were associated either with diet quality, or with meeting PA guidelines. Only higher property values did predict better diets and more physical activity. Better diets and more physical activity were associated with lower obesity prevalence at baseline and 12 mo, but did not predict weight change.

**Conclusion:**

Any links between the BE and health outcomes critically depend on establishing appropriate behavioral pathways. In this study, home-centric BE measures, were not related to physical activity or to diet. Further studies will need to consider a broader range of BE attributes that may be related to diets and health.

## Background

The relation between neighborhood built environment (BE) and obesity has been described as both nuanced and complex [1]. The largely cross-sectional literature has provided mixed results [1–4], such that establishing causal links between BE variables and obesity rates continues to be a challenge [1, 5–10]. Researchers have pointed to the need for more pathway-based analyses to examine associations between BE variables and diet quality and physical activity (PA) at the individual level. Any links between neighborhood BE and obesity are likely to involve diets, PA, or both.

Studies on the food environment have tended to focus on access to supermarkets, fast food restaurants, and convenience stores [1]. The access metrics were formulated in terms of presence/absence, counts, or densities within a certain buffer of home [3, 11–14]. Physical distance from home to the nearest supermarket was one metric of food access [2, 3, 7, 15–28]. Among other metrics were the number of and distances to supermarkets, grocery stores, fast food restaurants, and convenience stores [2, 17, 29–35]. Among PA-relevant metrics were the number of and distances to parks and recreational facilities, length of streets and sidewalks [26, 36–43].

The results were inconsistent. First, distance between the home and food sources was not always associated either with diet quality or with body weight [7, 44–49]. Some studies found that people living closer to supermarkets had lower body weights [2, 3, 7, 19, 26, 50–52] but other studies did not [46, 49, 53]. Some studies found that people living closer to fast foods and convenience stores were more likely to be obese [50, 54–57] but other studies did not [41, 46, 48]. One limitation of using home-centric proximity measures is physical closeness need not predict utilization. Only one-third of people in the Seattle Obesity Study I (SOS I) shopped at the nearest supermarket, and only about 10 % within their own census tract [49]. There are inherent limitations to the continued use of home-centric measures of exposure.

Perhaps more important, studies on neighborhood BE in relation to obesity prevalence often lacked data on diets or physical activity. The need to study the intermediate behavioral variables in the BE-obesity pathway has been stressed before [1, 7]. Combining data on neighborhood BE, dietary and PA behaviors and measured heights and weights at two points in time, the present study was able to ask and answer the following questions. First, what was the relation at baseline between some key BE variables within an 800 m buffer of home and diet and physical activity? Second, was there a relation between better diets and more physical activity at baseline and lower obesity prevalence, also at baseline, adjusting for covariates? Third, was there a relation between diet and PA at baseline and weight change over 12 months, adjusting for baseline weights? Unlike many past studies in this area, the SOS II was a longitudinal cohort, permitting us to examine the impact of BE on diets, physical activity and weight outcomes at baseline and at follow up.

## Methods

### Participant recruitment and data collection

A stratified, address-based sampling scheme was used to ensure spatial and economic distribution for the sample [58]. Residential units from about 450,000 tax parcels within King County Urban Growth Boundary (UGB) served as the sampling frame, tracking the county distribution of 58 % single family units and 48 % multifamily units. Units in three bands of residential property values (<199 K; > = 200 K- < 299 K; and > =300 K) were weighted to provide equal distributions [9, 58]. The reverse telephone matching of addresses with telephone numbers was conducted by a commercial supplier. The matching rate was 55 % for single and 40 % for multifamily units. After eliminating duplicates and incomplete records, 25,460 addresses and phone numbers were provided to the Battelle Memorial Institute Survey Research Group for telephone screening of potential study participants.

Battelle mailed out pre-notification postcards and followed with up to 13 telephone calls. Eligible participants were English speaking, aged 21–55y, were primary food shoppers in their household, and did not have mobility issues. After excluding non-working and business numbers, unreachable, ineligible participants and refusals, a list of 712 recruited participants was sent to the SOS II research staff to start with data collection procedures.

Eligible persons were contacted by phone and were invited to an in-person meeting. Of the eligible participants, 516 (72.5 %) agreed to enroll in the study. To minimize response bias and to include working mothers, single parents, and lower-income groups, all participants were given the choice of location for the first meeting: the UW or another location of their choice, including at his/her home. About 56 % of study participants (*n* = 291) chose the latter option. At the first in-person visit, participants provided written consent before data collection and were then weighed and measured. Participants were compensated with a monetary incentive of $100 for successful completion of baseline protocols, and another $100 for completing the follow up phase of the study. Five hundred sixteen respondents successfully completed the baseline phase of the study, and 478 completed the 1-year follow up phase of the study. However, after excluding those respondents with missing data on the variables of interest, the analytical sample consisted of 387 adults. The study protocols were approved by the Institutional review board (IRB) at the University of Washington. The study sample was recruited from Nov 2011–Oct 2013. The data were analyzed in the year 2015.

The study completes were comparable to Seattle-King County population in terms of income, education, and the prevalence of obesity and diabetes. As the study was restricted to ages 21–55y and gatekeepers, the sample was skewed towards younger population and females.

### The Health Behaviors Survey (HBS)

A computer-aided questionnaire was administered during the first visit. Data were collected on socio-demographics, and a variety of dietary, physical activity and health behaviors. Many of the questions were based on the Behavioral Risk Factors Surveillance System (BRFSS).

### Sociodemographic variables

Demographic variables were age, gender, race/ethnicity, household size, and home ownership. Annual household income and highest education level attained were the SES measures. For analyses, annual household income was grouped into three categories: low (<$50,000/y), medium ($50 K - < $100 K per year) and high (≥ $100 K per year). The education variable was dichotomized into: high school or some college (defined as <16y of education) vs. college graduates or higher (≥16y).

### Meeting Physical Activity Guidelines (PAG)

Participants were asked to report the frequency of moderate and vigorous physical activity per week along with duration at each. The CDC definition of physical activity was adapted for the present study. Those respondents who did moderate physical activity for 30 min per day for at least 5 days a week, or did vigorous activity for 20 min per day for at least 3 days a week, or did both were considered to meet PAG, versus the rest. For analytical purpose, PAG was treated as a dichotomous variable.

### Dietary variables: FFQ

The GNA version of Food Frequency Questionnaire (FFQ) developed by the Fred Hutchinson Cancer Research Center (FHCRC) has been used previously in large scale studies [59, 60]. Participants recorded the frequency of consumption of the listed foods and beverages, along with portion size. Completed FFQs, returned to the investigators, were checked for errors, stripped of all identifiers and sent to Nutrition Shared Resource at the FHCRC for processing. All the 387 respondents provided FFQ data. Nutrient composition analyses of their dietary intake data yielded dietary energy (kcal), the weight of foods, beverages, and drinking water (g), and the estimated daily intakes of over 45 macro- and micronutrients [61]. These data were also used to compute HEI-2010 scores for each respondent.

HEI-2010 is a continuous energy-adjusted measure of conformance to the 2010 Dietary Guidelines for Americans [62]. The measure consists of 12 components: nine adequacy and three moderation components including both foods and nutrients. Adequacy scores (with higher scores reflecting higher consumption) were total vegetables (five points), greens and beans (five points), total fruit (five points), whole fruit (five points), whole grains (ten points), dairy (tenpoints), total protein foods (five points), seafood and plant proteins (five points) and ratio of polyunsaturated and monounsaturated fatty acids to saturated fatty acids (ten points). Moderation scores (higher scores indicating lower consumption) included refined grains (ten points), sodium (tenpoints) and energy from solid fat, alcohol and added sugars (SoFAAS) (20 points). Energy from SoFAAS is a summary measure of “empty calories” or discretionary calories. The maximum HEI-2010 score is 100 points, with higher scores reflecting better diets.

### Measured heights and weights

Weight was measured in street clothes, without shoes, using a portable scale with a capacity of 350lbs. Two participants (out of 440) exceeded the 350 lb limit; their weight was recorded as 350 lb. Height was measured using a portable stadiometer. For analyses, objective weight and height data were converted to BMI (weight/height^2^), which was used to categorize respondents into obese (BMI ≥ 30 kg/m^2^) and non-obese (BMI <30).

### Built environment attributes

Several BE attributes were used and tested in 400 and 800 m buffers to define the respondents’ home neighborhood [63]. First, home addresses were geocoded using Arc ArcMap 10.2 (ESRI, Redlands, CA) to match respondents’ residential addresses to King County’s address point shapefile. Using a 100% matching criterion, 97.6 % (506 of the 516 SOS II baseline respondents) addresses were matched. Second, assessed property values were obtained from 2012 King County tax assessor parcel-level data, which contain separate values for the land and for the improvements attached to the land (i.e., structures and buildings). The assessed value per residential unit was calculated as the sum of a parcel’s land and improvement values divided by the number of residential units on the parcel. Mean neighborhood property values were then computed for all properties within the 400 and 800 m buffers of the respondent’s home. This individual-level metric is now established as an objective measure of neighborhood SES and detailed methodology has been published elsewhere [58]. For analytical purpose, the mean neighborhood values were split into tertiles. Fourth, permit data for food establishments, obtained from Public Health Seattle-King County (PHSKC), were classified into food establishment categories such as supermarkets, convenience stores, full service restaurants, fast-food or quick service restaurants, as previously described [64]. Food establishments were measured by presence or absence, and density within 400 and 800 m of participants’ homes.

Fifth, BE attributes related to physical activity included parks and trails often associated with physical activity [41, 65, 66], and length of streets and sidewalks that are related to neighborhood walkability [67, 68]. For parks, the UFL created a comprehensive database for King County, collected from each of the county’s 39 jurisdictions. Two analytical variables were used: the presence (≥3 parks) or absence (≤2 parks) of a park, and the number of parks within 400 and 800 m of respondents’ home. These cut offs were chosen based on the distribution of the present data for each buffer. Length of streets within a buffer is a measure of street density, which is itself correlated with intersection density. The longer the street lengths, the smaller the blocks, and greater is the intersection density. Small blocks and greater intersection density make a neighborhood more walkable.

Streets came from King County’s Metro Transportation Network data. For sidewalks, UFL assembled data from all the jurisdictions in King County and linked them to the street segment data. Details on assembly of this dataset are previously published [69]. ArcMap 10.2 was used to calculate the length of streets and the length of streets with full sidewalk coverage on either side within the 400 and 800 m buffers, these continuous variables were split into tertiles for analysis.

All the BE measures were developed at baseline. The authors anticipate minimal changes in BE measures such as changes in parks, streets or sidewalks over 12-month follow up period in the study area. There were changes in assessed property values but those were spread evenly across the County.

### Statistical analysis

All the BE variables were either dichotomized (yes or no), or split at median or converted into tertiles to best fit the distribution of the data. Both 400 and 800 m buffers for each variable were studied for data distribution. The 400 m buffer turned out to be too small to capture any variability in the present data, hence, all further analyses were conducted using 800 m buffer as the primary independent variables. HEI-2010 scores and % meeting PAG were the primary dependent variables. HEI - 2010 was tested as a continuous and as a dichotomous variable with median split. PAG was treated as a dichotomous variable. Prevalence of obesity at baseline and change in BMI at 12-months follow up were the health outcome variables.

Descriptive analyses such as chi^2^ tests were conducted to examine the distribution of health behaviors (i.e. diet quality and physical activity) by key socio-demographic and BE indicators. A series of regression analyses were conducted to examine each of the research questions. First, the association between BE indicators at baseline (using 800 m buffer) was examined in relation to health behaviors at baseline, using bivariate and multivariate regression analyses. HEI-2010 and proportion meeting physical activity guidelines (PAG) were each treated as dependent variables, and each of the BE indicators served as the independent variables separately. In multivariate models, the covariates were age, gender, race/ethnicity, income and education. Second, we tested if health behaviors at baseline (HEI-2010 and PAG each) were in turn associated with obesity prevalence at baseline using multivariate logistic regression analyses. HEI variable was analyzed as both a continuous and as a dichotomous variable. PAG was the dichotomous variable. Obesity prevalence was the dependent variable. Multiple models were conducted. Model 1 tested the associations of each – HEI and PAG with prevalent obesity, adjusting for age, gender, race/ethnicity, income, education, and living in Seattle vs. outside. Model 2 tested if the observed associations persisted after mutually adjusting for HEI and PAG in the model. Third, we further examined if HEI and PAG at baseline predicted change in BMI over 12-month follow up period. Due to lack of power with the incidence of obesity variable, change in BMI over 12-months was used as the primary dependent variable. The covariates were age, gender, race/ethnicity, income, education, living in Seattle vs. outside, and baseline BMI status.

Additional adjusted analyses tested the socioeconomic gradient in HEI-2010, PAG and obesity status in this cohort. Residential property values, income and education at baseline were the SES indicators. All the analyses were conducted using STATA 14.0. *P*-value of <0.05 was treated as statistically significant.

## Results

Table 1 shows the characteristics of the SOS II study sample. The majority of the participants were aged <50 years (61 %), were women (70.5 %), and Whites (83.2 %). Annual household incomes were mostly within 50 K-100 K range (37 %), with 34.9 % of the sample with incomes >100 K. The sample was college educated (66.7 %) and 77.2 % of participants owned their home. Most participants lived within 800 m of a fast food or convenience store; fewer were within 800 m of a park or a supermarket. In terms of health outcome indicators, almost half of the sample (45 %) lost >0.5Kg body weight during 12-months follow up, and 38 % gained at least 0.5 Kg weight. The mean change in BMI (follow up – baseline) was −0.2 Kg/m^2^ (SD: 1.5). The obesity prevalence at baseline was 32 %, which declined to 30 % by the end of 12-months. There were only eight incident cases of obesity, and 23 of the obese participants at baseline became non-obese by the end of 12-months.

**Table 1:** Distribution of diet quality and physical activity by socio-demographic and key environmental variables among SOS II participants

Characteristics	Total		HEI-2010 score ≥ median (73)		Meet PA guidelines (PAG)	
	N	%	N	%	N	%
All	387		194	(50.1)	228	(58.9)
Socio-demographics						
Age (years)						
21 - <50	236	(61.0)	116	(59.8)	138	(60.5)
50–55	151	(39.0)	78	(40.2)	90	(39.5)
Gender						
Women	273	(70.5)	141	(72.7)	154	(67.5)
Men	114	(29.5)	53	(27.3)	74	(32.5)
Race/ethnicity						
Whites	322	(83.2)	**172**	**(88.7)**	191	(83.8)
Non Whites	65	(16.8)	**22**	**(11.3)**	37	(16.2)
Household income						
< 50 K	108	(27.9)	**37**	**(19.1)**	**61**	**(26.8)**
≥ 50 K - <100 K	144	(37.2)	**78**	**(40.2)**	**75**	**(32.9)**
≥ 100 K	135	(34.9)	**79**	**(40.7)**	**92**	**(40.4)**
Education						
HS or some college (<16y)	129	(33.3)	**45**	**(23.2)**	**67**	**(29.4)**
College graduates or higher (≥16y)	258	(66.7)	**149**	**(76.8)**	**161**	**(70.6)**
Home ownership						
Own	299	(77.3)	**162**	**(83.5)**	176	(77.2)
Rent	88	(22.7)	**32**	**(16.5)**	52	(22.8)
BE measures (800 m buffer)						
Supermarket						
No	234	(60.5)	122	(62.9)	145	(63.6)
Yes	153	(39.5)	72	(37.1)	83	(36.4)
Convenience store						
No	137	(35.4)	72	(37.1)	86	(37.7)
Yes	250	(64.6)	122	(62.9)	142	(62.3)
Fast food/ QSR						
No	120	(31.0)	59	(30.4)	75	(32.9)
Yes	267	(69.0)	135	(69.6)	153	(67.1)
Park						
No	200	(51.7)	93	(47.9)	117	(51.3)
Yes	187	(48.3)	101	(52.1)	111	(48.7)

Numbers in brackets indicate column %s. Figures in bold indicate statistical significance based on chi2 tests (*p*-value <0.05)

Median HEI 2010 scores for the sample was 73. HEI scores rose with education, incomes, and home ownership and were higher for whites than non-whites. HEI 2010 scores were not associated with food sources in univariate analyses. In terms of PAG, 58.9 % of the sample met the guidelines. Higher income and education groups were more likely to meet PAG. No significant associations were observed by age, gender, race, and any of the BE attributes.

Table 2 shows the multivariate associations between BE attributes at baseline with HEI-2010 scores and meeting PAG at baseline. For these analyses, HEI 2010 was treated as a continuous variable, and PAG as the dichotomous variable. The data for HEI 2010 are therefore presented as coefficients and those for PAG as odds ratios. First, none of the food environment metrics showed any association with HEI, adjusting for sociodemographic variables. Similarly, none of the physical activity environment attributes were linked to PAG. Higher neighborhood property value was the only BE attribute linked to both higher HEI-2010 scores (β: 2.76 units for tertile 3; 95 % CI: 0.26, 5.26), and higher odds of meeting PAG (OR: 1.91 for tertile 3; 95 % CI: 1.10, 3.31).

**Table 2:** Multivariate associations of BE indicators (food and physical activity environment) at baseline in relation to diet quality and physical activity at baseline

Built environment within 800 m of home	HEI-2010			Physical activity (meet PAG)		
	Total			Total		
	Coef.	*P*-Value	95 % CI	OR	*P*-Value	95 % CI
Supermarket						
No	ref			ref		
Yes	−0.19	0.847	(−2.13, 1.75)	0.69	0.094	(0.45, 1.06)
Convenience store						
No	ref			ref		
Yes	−0.25	0.805	(−2.25, 1.75)	0.80	0.325	(0.51, 1.25)
Fast food/ QSR						
No	ref			ref		
Yes	0.48	0.646	(−1.57, 2.53)	0.81	0.371	(0.50, 1.29)
Park						
No (<=3)	ref			ref		
Yes (>3)	0.86	0.374	(−1.04, 2.77)	0.95	0.805	(0.62, 1.45)
Number of supermarkets						
Tertile 1 (0)	ref			ref		
Tertile 2 (1)	0.08	0.946	(−2.11, 2.26)	0.76	0.285	(0.45, 1.26)
Tertile 3 (2–4)	−0.58	0.694	(−3.51, 2.33)	0.61	0.091	(0.34, 1.08)
Number of convenience stores						
Tertile 1 (0)	ref			ref		
Tertile 2 (1–2)	−0.41	0.728	(−2.71, 1.89)	0.87	0.592	(0.53, 1.44)
Tertile 3 (3–16)	−0.05	0.965	(−2.43, 2.32)	0.72	0.227	(0.42, 1.23)
Number of fast foods/QSR						
Tertile 1 (0–1)	ref			ref		
Tertile 2 (2–8)	1.45	0.212	(−0.83, 3.75)	0.88	0.641	(0.52, 1.49)
Tertile 3 (9–157)	0.82	0.502	(−1.59, 3.24)	0.89	0.674	(0.53, 1.51)
Number of parks						
Tertile 1 (0–2)	ref			ref		
Tertile 2 (3–5)	**2.89**	**0.009**	**(0.72, 5.06)**	1.03	0.902	(0.64, 1.67)
Tertile 3 (6–16)	1.80	0.197	(−0.93, 4.54)	1.04	0.899	(0.59, 1.83)
Length of streets						
Tertile 1 (23 K - 71 K feet)	ref			ref		
Tertile 2 (72 K - 96 K feet)	0.92	0.435	(−1.39, 3.24)	1.18	0.518	(0.71, 1.96)
Tertile 3 (97 K - 158 K feet)	1.36	0.272	(−1.07, 3.81)	1.47	0.159	(0.86, 2.5)
Length of streets with sidewalks						
Tertile 1 (0 - 26 K feet)	ref			ref		
Tertile 2 (26 K - 67 K feet)	1.27	0.286	(−1.07, 3.62)	0.92	0.740	(0.55, 1.52)
Tertile 3(67 K - 124 K feet)	1.73	0.148	(−0.61, 4.08)	1.43	0.187	(0.84, 2.45)
Mean property value 800 m						
Tertile 1 (58 K - 275,443)	ref			ref		
Tertile 2 (275,468 - 383 K)	2.15	0.082	(−0.27, 4.59)	1.50	0.129	(0.89, 2.52)
Tertile 3 (383 K - 1245 K)	**2.76**	**0.030**	**(0.26, 5.26)**	**1.91**	**0.022**	**(1.10, 3.31)**

Adjusted for age, gender, race, income and education. Figures in bold indicate statistically significant values (p-value <0.05)

Table 3 shows multivariate associations of HEI 2010 scores, and PAG at baseline with prevalent obesity at baseline, and change in BMI over 12 months. In terms of prevalent obesity at baseline, every unit increase in HEI score was associated with significantly lower odds of obesity at baseline (OR: 0.97, 95 % CI: 0.95, 0.99), adjusting for key demographic variables (Model 1). These associations remained unchanged even after adding physical activity to the model (Model 2). Similar but stronger results were observed between PAG and obesity. Persons who met PAG had significantly lower odds of being obese at baseline (OR: 0.43, 95 % CI: 0.27, 0.69), adjusting for covariates (Model 1). This association attenuated slightly but remained significant after adding diet quality to the model (OR: 0.46, 95 % CI: 0.28, 0.74) (Model 2). Additional multivariate analyses with HEI-2010 defined as the dichotomous variable yielded the same results (Table 3). Respondents with HEI score of > =73 (median cut off for the present sample) had almost 50 % lower odds of being obese at each time point (OR: 0.52, 95 % CI: 0.32, 0.85 at baseline, and OR: 0.45 95 % CI: 0.27, 0.75 respectively), adjusting for covariates.

**Table 3:** Multivariate associations of diet quality and physical activity with prevalent obesity at baseline, and change in BMI over 1y follow up

	Prevalent obesity at baseline^a^						Change in BMI at 1-year follow up^b^					
	Model 1			Model 2			Model 1			Model 2		
	OR	*P*-value	95 % CI	OR	*P*-value	95 % CI	Coeff	*P*-value	95 % CI	Coeff	*P*-value	95 % CI
HEI-2010 (continuous)	**0.97**	**0.006**	**(0.95, 0.99)**	**0.97**	**0.025**	**(0.95, 0.99)**	−0.01	0.325	(−0.02, 0.01)	−0.01	0.338	(−0.02, 0.01)
Meets PA guidelines												
No	ref			ref			ref			ref		
Yes	**0.43**	**0.000**	**(0.27, 0.69)**	**0.46**	**0.001**	**(0.28, 0.74)**	−0.04	0.790	(−0.35, 0.27)	−0.03	0.875	(−0.34, 0.29)
HEI-2010 (median split)												
Below median	ref			ref			ref			ref		
Above median	**0.48**	**0.003**	**(0.30, 0.78)**	**0.52**	**0.009**	**(0.32, 0.85)**	0.11	0.495	(−0.21, 0.43)	0.11	0.485	(−0.20, 0.44)
Meets PA guidelines												
No	ref			ref			ref			ref		
Yes	**0.43**	**0.000**	**(0.27, 0.69)**	**0.45**	**0.001**	**(0.28, 0.73)**	−0.04	0.790	(−0.35, 0.27)	−0.05	0.754	(−0.37, 0.26)

^a^Model 1: adjusted for age, gender, race, income, education, living in Seattle vs. outside. Model 2: Model 1 + diet quality + physical activity

^b^Model 1: adjusted for age, gender, race, income, education, living in Seattle vs. outside, and baseline BMI status. Model 2: Model 1 + diet quality + physical activity

Figures in bold indicate statistical significance at different p-values

In terms of change in BMI longitudinally, neither HEI-2010 scores, nor physical activity at baseline predicted change in BMI over 12-months period in this study (Table 3).

Appendix examined the socioeconomic gradient in health behaviors (HEI and PAG) at baseline, obesity prevalence at baseline, and change in BMI longitudinally, adjusting for covariates. Higher residential property values and higher education at baseline were each associated with higher HEI-2010 scores at baseline (β: 3.32 in tertile 1, 95 % CI: 0.93, 5.72; and β: 4.25, 95 % CI: 2.17, 6.34 respectively), adjusted for demographics (Model 1). Higher income was also associated with significantly higher HEI scores at baseline (β: 2.82 in > =100 K, 95 % CI: 0.24, 5.40) (Model 2). Higher residential property value and higher education at baseline showed inverse association with obesity prevalence at baseline. However, none of these SES indicators were neither associated with physical activity at baseline, nor did these predict change in BMI over 12 months longitudinally.

## Discussion

**Fig. 1:**
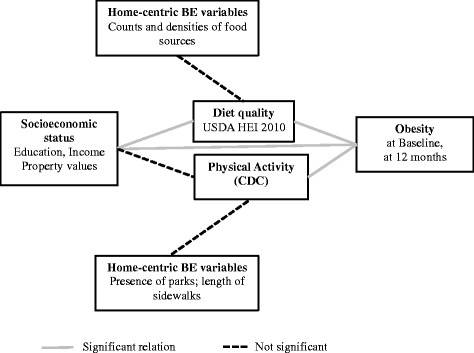
A schematic diagram of the observed relations among socioeconomic status, environment, behavior and health outcomes

Any links between neighborhood BE and obesity must involve behavioral pathways [1]. The present study is one of the first in this direction. None of the conventional BE indicators were found to be linked with health behaviors at baseline in this study. The only exception was residential property values, which showed significant associations with both diet quality and physical activity at baseline. In terms of health outcomes, both diet quality and physical activity were significantly linked with prevalent obesity at baseline, however, none of these predicted changes in BMI longitudinally (Fig. 1).

In the past, attempts to link access to supermarkets, grocery stores, convenience stores and fast foods directly with body weights have produced mixed results [1, 57]. Some studies found the expected associations, whereas many others did not [70–73]. Attempts to link access to food sources with diet quality measures were likewise mixed [74, 75]. While lower distance to the nearest supermarket was linked to better diets in some (but not all) studies [20, 38, 40, 76], distance to fast food restaurants had no consistent impact on diet quality [45, 46, 72]. A recent study of Los Angeles county adults failed to find links between the density of fast foods and convenience stores and BMI. One explanation was that motorized transport made proximity metrics irrelevant, at least in Los Angeles County [77].

By contrast, there is a consensus that PA-relevant neighborhood BE measures are associated with more physical activity [37, 78, 79], Specifically, streets, sidewalks and parks were found to support walking. In a study of 6376 same-sex adult twin pairs, higher neighborhood walkability was associated with more walking and lower BMI. These studies [80, 81] suggested that neighborhood walkability promoted walking and so reduced obesity risk.

The present analyses were based on some of the best-studied neighborhood BE measures, framed in terms of counts and densities of food sources and counts and densities of PA sources, including parks. In some prior studies, these or very similar measures were shown to have an impact on diet quality or diet composition [20, 76] and on PA measures [38, 40], respectively. However, no significant association between the conventional neighborhood BE metrics, as captured within a 800 m buffer of home, and diet quality or PA measures was observed in the present study. There was one BE variable that remained robustly linked with both diet quality and PA after adjusting for covariates. That variable was residential property values, rarely used in studies of the built environment and health.

The present null finding using conventional BE metrics raise a number of questions. First, did the BE variables used accurately reflect the multiple aspects of the neighborhood food and PA environments. For example, the nearest food store may not be the destination store where study participants actually shop for food. Studies of actual food destinations found that most people did not shop at the nearest supermarket, but rather went elsewhere [47, 49]. Though based on counts and densities, the results are consistent with prior reports that distances to food sources did not affect diet quality [4, 44, 45, 49, 53, 82]. It may be time to move from home-centric BE measures to GPS-based tracking of food activity space [83, 84].

GPS-based methods of assessing exposure to the BE beyond the home neighborhood may better capture how people interact with the BE in actual time and space [85–92]. This emerging work on “activity space” promises to bring information about where people shop for food and are physically active, and how their shopping and activity patterns might be influenced by BE beyond that of the home environment. Combined with measures of economic access to the BE, these methods will advance the understanding of causal paths to obesity.

Second, the PA-related BE measures may not have captured all the nuances of the PA environment. A recent study based on 6822 adults showed that residential density, intersection density, and the number of parks within 500 and 1000 m street network buffers (similar to the ones used here) were each associated with more PA [93]. The present study used number of parks, length of streets and streets with sidewalks as indices of walkability. However, no significant effects were observed.

There was one BE variable that did show a strong and consistent relation to diet quality and PA. Residential property values at tax parcel level, used here as an objective measures of individual and area SES, remained robust after adjusting for covariates. In reflecting on whether we selected the right BE variables, we considered the possibility that many of the aspects of neighborhood BE may be reliably captured by neighborhood property values. For example, Hunter et al. [94], writing on the relation between urban green spaces and physical activity, noted the desirability of wooded areas, open spaces, water features and pleasant views for PA promotion. Arguably, all those features might also be reflected in the value of the neighborhood real estate. In the present sample, as in other studies [95], obesity prevalence was strongly and inversely linked to residential property values at the tax parcel level.

A recent study suggests that distance to stores had less impact on diet quality than did differences in food purchasing power between the rich and the poor [96]. It may be time to consider the economic and other dimensions of food access and PA. When shoppers have access to a car, the distance between home and the grocery store is not the limiting variable. Proximity to parks or waterfront may capture socioeconomic status but little else. There is need to consider economic – rather than physical – access to healthy food and an environment which permits physical activity.

Consistent with those observations, the present results showed a cross-sectional links between better diets and more PA at baseline and lower obesity prevalence. Those associations held for obesity measured at baseline and for obesity measured at 12 month follow up. Similar links between better diets and lower obesity rates have been made in other studies. However, a follow up analysis of obesity risk at 12 months, adjusting for baseline weights, did not show a significant effects of baseline diets or PA. In another study, we found that obesity rates at baseline were strongly and inversely linked to SES; however, SES had no impact on 12-month weight change. Weight trajectories may be driven by individual motivations rather than by SES or baseline diet. However, it may be that the study did not have enough power. There were only eight incident obesity cases in the sample.

The present study advances the literature in a number of ways. First, neighborhood BE attributes were objectively measured in GIS. Second, diet quality was measured using the Healthy Eating Index 2010 (HEI - 2010). The HEI - 2010 was developed by the US Department of Agriculture to assess compliance with the 2010 Dietary Guidelines. Third, obesity prevalence was based on measured heights and weights, obtained at baseline and at 12-month follow up. Prospective longitudinal cohorts have rarely been used in studies of BE and health outcomes. Cross-sectional study designs, the mainstay of the existing literature on the BE and health, do not permit the drawing of causal inferences.

Limitations included the relatively small cohort and short 12-month exposure period, when no change in the BE could be observed. Self-selection into neighborhoods, always a potential confound, may have been driven by the availability and type of food or physical activity environment in the neighborhood, or by home prices. Dietary intakes and physical activity were collected by self-report. The home neighborhood may be a too restrictive geographic extent as respondents’ behaviors may be influenced by environments that lie beyond their immediate surrounds, including their work or school environment. Finally, because the characteristics of neighborhoods differ between cities, the results of this study may not be generalizable to settings outside of Seattle–King County.

## Conclusion

Selected conventional measures of neighborhood BE had no impact on diet quality, PA, or on obesity rates. By contrast, diet quality, PA, and obesity rates were each strongly linked to neighborhood property values. Furthermore, better diets and more physical activity were linked to lower obesity prevalence at baseline but did not predict 12-month weight change. Future studies on the impact of BE on health need to consider the importance of socioeconomic variables known to influence residential decisions.

## Appendix

**Table 4:** To examine the socioeconomic gradient in diet quality, physical activity, and prevalent obesity at baseline, and change in BMI over 1y follow up

N (%)	HEI-2010^a^			Meets PA guidelines^a^			Prevalent obesity at baseline^a^			Change in BMI at 1 year follow up^b^		
	Coef.	*P*-value	95 % CI	OR	*P*-value	95 % CI	OR	*P*-value	95 % CI	Coeff	*P*-value	95 % CI
Model 1												
Property value												
Tertile 1 (38 K - 231 K)	**ref**			ref			**ref**			ref		
Tertile 2 (233 K - 331 K)	1.92	0.115	(−0.47, 4.32)	0.91	0.712	(0.55, 1.50)	1.17	0.549	(0.69, 1.97)	−0.03	0.859	(−0.42, 0.32)
Tertile 3 (338 K - 914 K)	**3.32**	**0.007**	**(0.93, 5.72)**	1.57	0.102	(0.91, 2.70)	**0.32**	**0.000**	**(0.17, 0.60)**	−0.13	0.526	(−0.55, 0.28)
Education												
≤ Some college	**ref**			ref			**ref**			ref		
≥ College 4 years	**4.25**	**0.000**	**(2.17, 6.34)**	1.38	0.164	(0.87, 2.18)	**0.51**	**0.006**	**(0.31, 0.82)**	−0.06	0.729	(−0.44, 0.31)
Model 2												
HH income^b^												
< 50 K	ref			ref			ref			ref		
≥ 50 K - < 100 K	1.95	0.127	(−0.55, 4.46)	0.81	0.423	(0.48, 1.35)	0.81	0.467	(0.47, 1.41)	−0.02	0.913	(−0.43, 0.38)
≥ 100 K	**2.82**	**0.032**	**(0.24, 5.40)**	1.52	0.132	(0.87, 2.66)	0.59	0.079	(0.33, 1.06)	0.27	0.130	(−0.08, 0.62)
Education												
≤ Some college	**ref**			ref			**ref**			ref		
≥ College 4 years	**4.39**	**0.000**	**(2.21, 6.56)**	1.37	0.410	(0.12, 2.31)	**0.46**	**0.002**	**(0.28, 0.74)**	−0.06	0.729	(−0.44, 0.31)

^a^Model 1: included age, gender, race/ ethnicity, residential property values and education. Model 2: included age, gender/ race/ethnicity, income and education

^b^Model 1: included age, gender, race/ ethnicity, residential property values, education and baseline BMI status. Model 2: included age, gender/ race/ethnicity, income, education and baseline BMI status. Figures in bold indicate statistically significant values (p-value <0.05)

## Abbreviations

BE: Built environment
BMI: Body mass index
CI: Confidence intervals
FFQ: Food frequency questionnaire
HEI: Healthy eating index
PA: Physical activity
PAG: Physical activity guidelines
SOS: Seattle obesity study
